# Coordinator Leadership in the Relationship Between Burnout and Nurses’ Intention to Leave: A Cross-Sectional Study

**DOI:** 10.3390/healthcare14070858

**Published:** 2026-03-27

**Authors:** Francesco Zaghini, Flavio Marti, Cesar Ivan Aviles Gonzalez, Marika Lo Monaco, Davide Bartoli, Mariachiara Figura, Giovanni Gioiello

**Affiliations:** 1 Department of Biomedicine and Prevention, University of Rome Tor Vergata, Via Montpellier 1, 00133 Rome, RM, Italy; francesco.zaghini@uniroma2.eu (F.Z.); flavio.marti@uniroma1.it (F.M.); 2 Department of Medicine and Surgery, University of Enna “Kore”, Piazza dell’Università, 94100 Enna, EN, Italy; cesar.aviles@unikore.it; 3 Department of Maternal and Child Health Promotion, Internal Medicine & Specialties of Excellence “G. D’Alessandro” (PROMISE), University of Palermo, Piazza delle Cliniche 2, 90127 Palermo, PA, Italy; marika.lomonaco@unipa.it (M.L.M.); mariachiara.figura@unipa.it (M.F.); 4 Interdisciplinary Department of Wellbeing, Health and Environmental Sustainability-BeSSA Department, Sapienza University of Rome, Piazzale Aldo Moro 5, 00185 Roma, RM, Italy; davide.bartoli@uniroma1.it

**Keywords:** nurses, burnout, leadership, intention to leave, work environment

## Abstract

**Highlights:**

**What are the main findings?**

- Interpersonal conflicts, workload, and organizational constraints are key work-related factors linked to burnout among nurses.
- Burnout is related to nurses’ intention to leave the profession, with ethical leadership of nurse coordinators emerging as a partial mediator in this relationship.

**What are the implications of the main findings?**

- Ethical leadership by nurse coordinators may represent an organizational resource linked to a weaker relationship between burnout and nurses’ intention to leave.
- Interventions targeting work environment conditions and leadership development may be linked to improvements in nurses’ well-being and workforce retention.

**Abstract:**

**Background/Objectives**: Nursing turnover represents an increasing threat to the sustainability of healthcare systems. Burnout, a syndrome of chronic work-related stress, is one of the primary predictors of intention to leave work; however, certain organizational factors may be associated with variations in its impact. Among these, the leadership of the Unit Coordinator may represent a potential resource, but its association with the relationship between burnout and intention to leave remains poorly explored. This study investigates the role of coordinators’ leadership in the relationship between burnout and intention to leave the profession. **Methods**: A cross-sectional study was conducted among 668 nurses providing direct patient care in various Italian healthcare settings. Data were collected through an online questionnaire comprising validated scales reported in the literature. A structural equation modeling approach was used for the analysis. **Results**: More than 30% of the variance in burnout is explained by interpersonal conflicts, workload, and organizational constraints. Burnout accounts for 24.4% of the variance in nurses’ intention to leave their jobs. The leadership of the nurse coordinator partially mediates the relationship between burnout and nurses’ intention to leave their job (total effect β = 0.532; *p* < 0.001; indirect effect β = 0.139; *p* = 0.007; direct effect β = 0.393; *p* < 0.001). **Conclusions**: Burnout is a key predictor of nurses’ intention to leave the profession, while ethical leadership of nurse coordinators emerges as a potential organizational resource associated with this relationship. **Nursing implications**: These findings highlight the importance of promoting ethical leadership within nursing management as part of broader organizational strategies to improve staff well-being and potentially support efforts aimed at reducing nurses’ intention to leave the profession.

## 1. Introduction

In recent years, the intention to leave the nursing profession has emerged as a major concern for healthcare systems worldwide. The World Health Organization [1] has repeatedly emphasized that the shortage of nursing personnel constitutes a serious threat to the sustainability and quality of healthcare services, particularly in contexts characterized by regional inequalities in workforce availability [2].

In this context, turnover intention (ITL)—that is, nurses’ stated willingness to leave their current position or the profession—represents a reliable predictor of actual departure from the profession [3,4].

Recent empirical evidence has shown that nurses’ intention to leave their hospital or the nursing profession is associated with several work-related and individual factors, including emotional exhaustion, depersonalization, dissatisfaction with work prospects, limited opportunities for professional development, and work-related health problems [5].

Among the factors influencing ITL, the literature identifies burnout as one of the most significant predictors. This psychological syndrome, arising from prolonged exposure to work-related stressors, is characterized by emotional exhaustion, depersonalization, and reduced personal accomplishment [6]. Its consequences are substantial at both the individual and organizational levels, leading to deterioration in mental and physical health, increased absenteeism, a decline in the quality of care, and a heightened propensity toward ITL [7,8,9].

Burnout among nurses is the result of complex and multifactorial determinants [10]. Among the most relevant contributors are interpersonal conflicts [11], often associated with dysfunctional relational dynamics among nurses, physicians, and other healthcare professionals [12]; high workloads [13], encompassing not only the intensity and frequency of required tasks but also their emotional burden; and, finally, organizational constraints [14,15], such as bureaucratic rigidity, resource shortages, and insufficient institutional support [16]. These conditions converge to create a work environment perceived by nurses as hostile and unrewarding, fostering a process that may progressively lead to ITL [17,18].

In this context, the relationship among organizational stressors, burnout, and turnover intention can be understood through resource-based occupational stress models, such as the Job Demands–Resources (JD-R) model [19,20]. According to this framework, demanding work conditions (such as excessive workload, interpersonal conflicts, or insufficient organizational support) consume employees’ psychological resources and increase the likelihood of burnout. Some studies have further confirmed that unfavorable working conditions and burnout-related symptoms play a central role in shaping nurses’ intention to leave their organization or the profession [21,22]. Conversely, organizational resources can buffer the negative impact of job demands and support employees’ well-being [19]. When job demands exceed available resources, employees may experience psychological strain that contributes to burnout and eventually increases their intention to leave the organization or profession [23].

Within this framework, leadership behaviors can also be conceptualized as important organizational resources that buffer the negative effects of job demands on employees’ psychological well-being [24], particularly in complex healthcare systems where nurse leaders must manage interdependent organizational, relational, and clinical dynamics [25]. These findings further highlight the importance of organizational resources (such as supportive and ethical leadership) in mitigating the impact of work-related stressors and reducing nurses’ intention to leave.

In such a scenario, characterized by a demanding organizational context that fuels burnout and, consequently, ITL, the leadership exercised by nursing coordinators can play a crucial role. The literature shows that ethical leadership can reduce burnout levels among workers [26], while fostering greater engagement [27] and commitment [28], including in nursing contexts [29,30].

From a theoretical perspective, ethical leadership may shape employees’ attitudes and behaviors by strengthening relational and psychological resources within the workplace. When leaders are perceived as ethical, employees tend to develop greater trust in management and improved psychological well-being [31].

By fostering fairness, trust, and supportive relationships within teams, ethical leadership may help preserve employees’ psychological resources and reduce emotional exhaustion. In this way, leadership behaviors may indirectly influence employees’ intention to leave by mitigating the resource depletion process that typically leads to burnout.

Empirical studies have shown that leadership behaviors may significantly influence nurses’ well-being and turnover intentions. For instance, ethical leadership has been associated with lower levels of workplace bullying, reduced burnout, and decreased turnover intention among nurses [26]. Similarly, leadership approaches that emphasize support, fairness, and collaboration may help employees cope with demanding work environments and maintain engagement with their work [32].

Evidence has shown that work environment and burnout mediate the relationship between authentic leadership and nurses’ intention to leave their job [33]. Similarly, burnout has been found to partially mediate the relationship between responsible leadership and turnover intention [34].

A coordinator who adopts an ethical leadership style, positioning themselves as a point of reference for the team, can mitigate the negative impact of a burdensome organizational environment by fostering a climate of trust, support, and fairness, ultimately contributing to lower burnout levels and reduced ITL among nurses [35].

Despite this evidence, the existing literature has mainly examined the direct effects of leadership or burnout on turnover intention, while the mechanisms through which leadership behaviors may indirectly influence turnover intention through burnout remain insufficiently explored. Few studies have specifically investigated the role of ethical leadership among nursing coordinators within the relationship between organizational stressors, burnout, and nurses’ intention to leave their jobs.

In the present study, ethical leadership was conceptualized as a mediating rather than a moderating mechanism. This choice was theoretically grounded in the Job Demands–Resources framework, which suggests that organizational resources influence employees’ psychological processes and outcomes. Specifically, ethical leadership may shape how burnout translates into turnover intention by affecting relational and psychological resources within the workplace. Thus, the model focuses on a process-oriented pathway rather than on interaction effects. However, alternative models (e.g., moderation or moderated mediation) may also be relevant and should be explored in future research.

Given the substantial impact of nurse turnover on healthcare quality and the sustainability of healthcare systems, a deeper understanding of these mechanisms appears particularly important. Clarifying the role of ethical leadership may help identify organizational leverage points to prevent burnout, reduce turnover intention, and improve nurses’ well-being.

In light of these considerations, the primary objective of the present study was to investigate the role of ethical leadership among nursing coordinators in the relationship between burnout and nurses’ intention to leave the profession.

**Figure 1:**
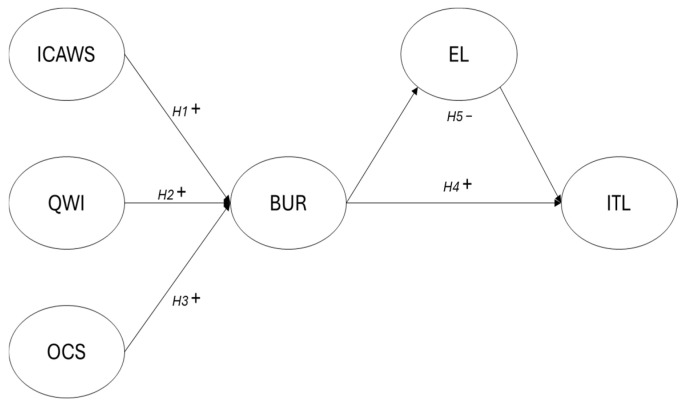
Hypothesized Model. Legend: ICAWS = Interpersonal Conflicts; QWI = Workload; OCS = Organizational Constraints; BUR = Burnout; EL = Ethical Leadership; ITL = Intention to leave.

As can be seen in Figure 1, the following hypotheses were tested:

- H1, H2, H3: Nurses’ burnout levels are explained by interpersonal conflicts (ICAWS), workload (QWI), and organizational constraints (OCS);
- H4: Nurses’ burnout explains the variability in their intention to leave work (ITL);
- H5: Ethical leadership style (EL) mediates the relationship between burnout and nurses’ intention to leave work (ITL).

## 2. Materials and Methods

This study adopted a cross-sectional, descriptive, and correlational design. To test the hypothesized model, a multicenter study was conducted among nurses working in different Italian healthcare settings.

### 2.1. Participants and Procedure

The study involved administering the questionnaire to all nurses assigned to the participating healthcare organizations’ operational units who met the predefined inclusion criteria. Specifically, nurses providing direct patient care, working both on shifts and off-shift schedules, and employed under any type of contract (e.g., full-time, part-time, fixed-term, or permanent) were recruited. Due to the questionnaire distribution method, it was not possible to determine the exact number of nurses who received the invitation; therefore, a response rate could not be calculated.

For data collection, the developed instrument was uploaded to an online platform, and a link was generated for distribution to participants. The link was distributed via emails sent to institutional accounts or through the regular communication channels of the participating healthcare organizations.

### 2.2. Instruments

Data were collected using a questionnaire composed of previously validated scales from the literature. The questionnaire consisted of several sections. The first section, visible upon opening the link, included an information sheet describing the study, its objectives, and the procedures for handling personal data. At the end of the information sheet, participants were asked to provide consent to participate in the study and to consent to the processing of personal data. After providing consent, participants could proceed to complete the subsequent sections of the questionnaire. These sections included the scales listed in Table 1, followed by a final section requesting sociodemographic and work-related information.

After conducting a pilot test of the questionnaire, it was verified that completion required no more than 15 min. The questionnaire remained available for 4 weeks.

All instruments were administered in their Italian versions. When available, previously validated Italian versions of the scales were used. No substantial modifications to the original items were made. For instruments originally developed in other languages, the Italian versions used in this study were consistent with those adopted in prior research in Italian healthcare settings, ensuring conceptual equivalence of the constructs.

### 2.3. Statistical Analysis

Data analysis was performed in several stages. First, descriptive analyses were conducted to summarize the sample characteristics and the variables under study, including means, standard deviations, frequency distributions, and percentages. The reliability of the scales used to measure the study variables was assessed using Cronbach’s alpha. Subsequently, Pearson’s correlation coefficient (r) was calculated to examine the relationships between the study variables.

Finally, to test the hypothesized model, a structural equation modeling (SEM) approach was employed. In the SEM analysis, all constructs were specified as latent variables, with each construct represented by its respective observed indicators (i.e., individual items of the scales). Model fit was considered acceptable based on the following indices: Chi-square (χ^2^) (non-significant), RMSEA (<0.06), CFI (>0.90), TLI (>0.90), and SRMR (<0.08) [45,46]. Indirect effects were tested using bootstrapped confidence intervals based on resampling procedures implemented in Mplus, which provides robust estimates of mediation effects.

Missing data were handled using different strategies depending on the type of analysis. Descriptive and correlation analyses were performed using the pairwise deletion method in SPSS, resulting in slight differences in sample sizes across variables. Structural equation modeling (SEM) analyses were conducted in Mplus using full information maximum likelihood (FIML), which allows the inclusion of partially observed data under the assumption of missing at random. Descriptive, correlation, and reliability analyses were performed using SPSS version 26, while SEM analyses were conducted using Mplus version 8.

### 2.4. Ethical Considerations

The study was conducted in accordance with the Helsinki principles [47] and was approved by the Ethics Committee of the University of Palermo (n. 157442 del 18 September 2025). Eligible nurses were informed about the study aims and procedures; participation was voluntary, and they could withdraw at any time. They were also informed that their data would be treated confidentially, and their identities would not appear in written records. Willing participants signed informed consent for study participation and data treatment in accordance with current privacy laws. The data were collected, processed, and analyzed in compliance with privacy and anonymity.

## 3. Results

As shown in Table 2, a total of 668 nurses participated in the study, predominantly female (76.3%), with a mean age of 42.4 years (SD = 10.73). Most participants held a bachelor’s degree (59.3%), followed by a regional diploma (27.7%) and a university nursing diploma (13.0%). On average, nurses in the sample had 17.26 years of work experience (SD = 10.57) and reported 9.9 monthly overtime hours (SD = 9.13). In the past six months, participants had taken an average of 6.72 sick leave days (SD = 14.40).

### 3.1. Correlations Among Study Variables

Descriptive analyses of the variables included in the study indicated moderate levels of interpersonal conflicts (M = 2.28; SD = 0.81), workload (M = 3.05; SD = 1.08), and organizational constraints (M = 2.57; SD = 0.82). Ethical leadership (EL) showed relatively high scores (M = 3.66; SD = 1.00), whereas burnout (M = 2.17; SD = 1.22) and turnover intention (M = 2.92; SD = 0.79) were at moderate levels. All measurement scales demonstrated good reliability (range = 0.70–0.96) (Table 3).

The correlation matrix indicates significant relationships among all variables (*p* < 0.01). In particular, ICAWS is positively correlated with QWI (r = 0.442, *p* < 0.001), OCS (r = 0.578, *p* < 0.001), BUR (r = 0.461, *p* < 0.001), and ITL (r = 0.243, *p* < 0.001), while it is negatively associated with ethical leadership (r = −0.340, *p* < 0.001). QWI and OCS are also positively associated with each other (r = 0.576, *p* < 0.001) and with BUR (respectively, r = 0.396 and r = 0.502, both *p* < 0.001). Ethical leadership shows inverse correlations with all stress-related variables, including ITL (r = −0.221, *p* < 0.001). Finally, intention to leave work is positively associated with all sources of stress, particularly burnout (r = 0.494, *p* < 0.001)

### 3.2. Structural Equation Model Results

**Figure 2:**
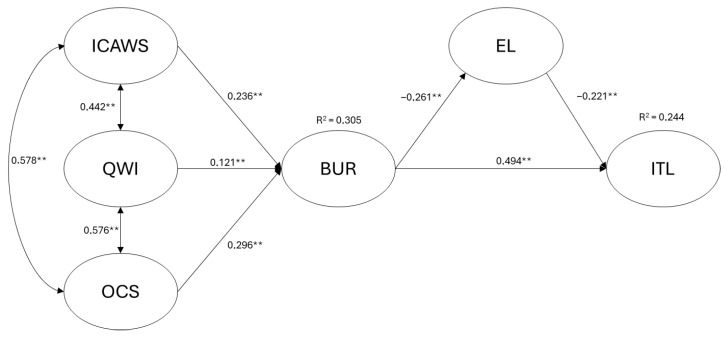
Results of the Tested Model. Note: ** = *p* < 0.001; ICAWS = Interpersonal Conflicts; QWI = Workload; OCS = Organizational Constraints; BUR = Burnout; EL = Ethical Leadership; ITL = Intention to leave.

The structural equation model, estimated using the MLr method, allowed us to examine the mediating effect of ethical leadership among nurse managers on the relationship between burnout and nurses’ turnover intention (ITL). The model showed an overall acceptable fit to the data. Specifically, CFI (0.958), TLI (0.940), and SRMR (0.043) met the recommended thresholds, while RMSEA (0.076) was slightly above the more stringent cut-off (<0.06), suggesting an acceptable but not optimal fit. As shown in Figure 2, the model indicated that over 30% of the variance in burnout (R^2^ = 0.305) is explained by interpersonal conflicts (H1; β = 0.236; *p* < 0.001), workload (H2; β = 0.121; *p* = 0.003), and organizational constraints (H3; β = 0.296; *p* < 0.001).

Burnout explains 24.4% of the variance in turnover intention (R^2^ = 0.244). The model includes a partial mediating effect of ethical leadership, with a total effect of β = 0.532 (*p* < 0.001), an indirect effect of β = 0.139 (*p* = 0.007), and a direct effect of β = 0.393 (*p* < 0.001).

## 4. Discussion

This study was designed with the primary objective of examining the role of nurse coordinators’ Ethical Leadership in the relationship between burnout and nurses’ intention to leave the profession. The need to understand this relationship arises within a healthcare context marked by high organizational complexity and a progressive increase in the risk of turnover among nursing staff [48], which represents one of the main critical issues for health systems worldwide, with direct consequences for quality of care, patient safety, and the sustainability of healthcare organizations [49].

Overall, the findings of this study allowed an examination of the relationships between specific dimensions of the work environment and the phenomenon of burnout, with particular attention to interpersonal conflicts (ICAWS—Interpersonal Conflict at Work Scale) (H1), workload (QWI—Quantitative Workload Inventory) (H2), and organizational constraints (OCS—Organizational Constraints Scale) (H3). The observed correlation patterns among the study variables are consistent with the theoretical model and support the expected relationships among work stressors, burnout, ethical leadership, and intention to leave. These factors were confirmed as key determinants of burnout among nursing staff, as widely acknowledged in the scientific literature [10,14]. In particular, the results show that these organizational dimensions explain more than 30% of the variance in burnout, highlighting the potential relevance of targeted interventions to improve working conditions and promote nurses’ well-being.

Consistent with previous literature [50,51], this study’s results confirm that burnout is a significant predictor of intention to leave the profession. Specifically, the findings indicate that burnout accounts for more than 24% of the variance in nurses’ intention to leave their job (R^2^ = 0.244), underscoring the substantial association between demanding organizational contexts and nurses’ career intentions. In a setting already characterized by severe staffing shortages [52] and an increasing care burden [53], this relationship emerges as particularly alarming [54].

Although burnout maintains a significant direct effect on nurses’ intention to leave the profession, an important aspect emerging from this study concerns the role of Ethical Leadership among nurse unit managers in this relationship. The findings suggest that Ethical Leadership is associated with a weaker relationship between burnout and intention to leave and may be linked to the promotion of a work climate characterized by support, listening, and dialogue [27,55].

These results should be interpreted as associative rather than causal, given the study’s cross-sectional design. This finding, which aligns with recent literature, recognizes Ethical Leadership as an important organizational resource potentially linked to the psychological and motivational well-being of nursing staff [31]; therefore, it must be implemented, cultivated, and promoted within organizations. Existing studies confirm that an ethical leadership style is associated with tangible positive outcomes, such as greater staff and patient satisfaction, increased productivity, and the spread of ethical behaviors among professionals [29]. Moreover, organizational contexts characterized by ethical leadership behaviors are associated with more equitable, supportive, and psychologically safe work environments, which in turn may be linked to staff performance and turnover reduction [26,56].

From a practical perspective, these findings highlight the importance of leadership development initiatives to strengthen ethical leadership competencies among nurse coordinators. Such initiatives may be associated with the creation of more supportive work environments and with improvements in the organizational climate in healthcare settings. Examples of such initiatives may include structured training programs focused on ethical decision-making, leadership coaching interventions, and organizational actions such as the implementation of shared governance models or formal support systems for staff.

However, further empirical evidence, particularly from intervention-oriented and longitudinal studies, is needed to better understand how leadership development initiatives may translate into measurable improvements in nurse well-being and workforce stability.

Recent intervention-oriented research suggests that organizational and educational strategies aimed at strengthening leadership practices and resilience may support psychological well-being and coping capacities within the nursing workforce, highlighting the potential value of preventive approaches across different stages of professional development [57,58].

### Limitations

The results of this study, while representing an important contribution to the scientific community, should be interpreted considering several limitations.

First, social desirability may have influenced participants’ responses to certain items, particularly those related to organizational constraints and the coordinator’s ethical leadership; therefore, the findings should be interpreted with caution.

Second, the complexity and variability of the organizational context must be taken into account, as it may be affected by multiple factors not included in the present model. For instance, emotional labor [59] could have influenced the results to an extent not captured by the study.

Another limitation concerns the cross-sectional study design, which does not allow for causal inferences between the variables examined, but only for the observation of associations. Furthermore, the cross-sectional nature of the data does not rule out reverse causality, particularly in the relationship between burnout and intention to leave.

In addition, exclusive reliance on self-report measures may introduce common method variance, potentially inflating the observed associations among the study variables. No formal statistical assessment of common method bias (e.g., Harman’s single-factor test or CFA marker techniques) was conducted; however, the use of validated instruments and the anonymity of responses may have partially mitigated this risk. Moreover, the inability to calculate a response rate prevents the assessment of potential non-response bias. Although missing data in SEM were handled using full information maximum likelihood (FIML), a widely recommended approach under the assumption of missing at random, no additional sensitivity analyses (e.g., multiple imputation or listwise deletion comparisons) were conducted. Therefore, the potential impact of alternative missing data handling strategies cannot be entirely excluded, although it is likely to be limited. Furthermore, the online data collection method may have affected response quality, as participants could have discussed the questions with colleagues, potentially introducing response bias. Although the large sample is a strength of the study, caution is warranted when assessing the generalizability of the findings. The data were collected within Italian healthcare contexts, and cultural, organizational, or systemic differences may limit the direct applicability of the results to other national healthcare systems.

## 5. Conclusions

This study examined the relationship between burnout and nurses’ intention to leave the profession and explored the potential role of ethical leadership among nurse coordinators within this relationship. The findings indicate that work-related stressors such as interpersonal conflict, workload, and organizational constraints are associated with higher levels of burnout among nurses. In turn, burnout is significantly associated with nurses’ intention to leave their job. The results also suggest that ethical leadership is related to this relationship, as it is associated with a weaker link between burnout and turnover intention.

These findings highlight the importance of organizational strategies to improve working conditions and strengthen leadership competencies among nurse coordinators in healthcare settings. Leadership development initiatives focused on ethical leadership may represent a promising area for organizational investment, based on these associative findings, in order to support positive work environments and staff well-being.

Future research should further investigate these relationships using longitudinal and multi-level study designs capable of examining temporal processes and organizational mechanisms underlying burnout and turnover intention among nurses.

## Figures and Tables

**Table 1:** Measurement Instruments.

Variable	Scale	Authors	N. of Items	Dimensions	Response Format
**Interpersonal Conflicts**	Interpersonal Conflict at Work Scale (ICAWS)	[36,37,38]	4	Single	5-point Likert scale (1 = “Never or Almost Never” to 5 = “Very Often/Always”)
**Organizational Constraints**	Organizational Constraints Scale (OCS)	[36,37,38]	9	Single	5-point Likert scale (1 = “Never or Almost Never” to 5 = “Very Often/Always”)
**Burnout**	Maslach Burnout Inventory–General Survey (MBI-GS) and Interpersonal Strain at Work (ISW)	[39,40,41,42]	15	3 (Emotional Exhaustion, Cynicism, Interpersonal Strain)	7-point Likert scale (0 = “Never” to 6 = “Always”)
**Ethical Leadership**	Ethical Leadership Scale (ELS)	[43]	10	Single	5-point Likert scale (1 = “Strongly Disagree” to 5 = “Strongly Agree”)
**Turnover Intention**	Turnover Intention Scale (TIS)	[44]	6	Single	5-point Likert scale (1 = “Never” to 5 = “Always”)

**Table 2:** Sociodemographic and work characteristics.

Variable	N	%	M	SD
Gender				
Female	510	76.3		
Male	158	23.7		
Age	665		42.40	10.73
Professional Title				
Bachelor’s degree	396	59.3		
Regional Certificate	185	27.7		
University Diploma in Nursing	87	13.0		
Work experience	665		17.26	10.57
Years in the organization	657		10.90	10.60
Overtime hours (monthly)	660		9.90	9.13
Absences (yearly)	667		6.72	14.40

**Table 3:** Correlation Matrix among Study Variables.

Variables	M	SD	α	ICAWS	QWI	OCS	BUR	EL
ICAWS	2.28	(0.81)	0.828					
QWI	3.05	(1.08)	0.894	0.442 **				
OCS	2.57	(0.82)	0.894	0.578 **	0.576 **			
BUR	2.17	(1.22)	0.955	0.461 **	0.396 **	0.502 **		
EL	3.66	(1.00)	0.936	−0.340 **	−0.218 **	−0.410 **	−0.261 **	
ITL	2.92	(0.79)	0.695	0.243 **	0.234 **	0.373 **	0.494 **	−0.221 **

Note: ** = *p* < 0.001; ICAWS = Interpersonal Conflicts; QWI = Workload; OCS = Organizational Constraints; BUR = Burnout; EL = Ethical Leadership; ITL = Intention to leave.

## Data Availability

The original contributions presented in this study are included in the article. Further inquiries can be directed to the corresponding author.

## Abbreviations

The following abbreviations are used in this manuscript:

BUR	Burnout
CFI	Comparative Fit Index
EL	Ethical Leadership
ELS	Ethical Leadership Scale
ICAWS	Interpersonal Conflict at Work Scale
ISW	Interpersonal Strain at Work
ITL	Intention to Leave
MBI-GS	Maslach Burnout Inventory–General Survey
OCS	Organizational Constraints Scale
QWI	Quantitative Workload Inventory
RMSEA	Root Mean Square Error of Approximation
SD	Standard Deviation
SEM	Structural Equation Modeling
SRMR	Standardized Root Mean Square Residual
TLI	Tucker–Lewis Index
